# Editorial: Emerging trends in phage therapeutics to overcome antibiotic resistance

**DOI:** 10.3389/frabi.2026.1788766

**Published:** 2026-02-24

**Authors:** William Calero-Cáceres, Ankush Gupta, Anju Kaushal, Paul Hyman

**Affiliations:** 1 UTA-RAM-One Health, Group for Universal Advance in bioScience, Department of Food and Biotechnology Science and Engineering, Universidad Técnica de Ambato, Ambato, Ecuador; 2 Biotechnology Program, Department of Food and Biotechnology Science and Engineering, Universidad Técnica de Ambato, Ambato, Ecuador; 3 Department of Biochemistry, Institute of Science, Banaras Hindu University, Varanasi, India; 4 Panjab University, Chandigarh, India; 5 New Zealand Organization for Quality, Auckland, New Zealand; 6 University of Auckland, Auckland, New Zealand; 7 Department of Biology & Toxicology, Ashland University, Ashland, OH, United States

**Keywords:** AMR (antimicrobial resistance), antibiotic resistance, bacteriophage, phage (bacteriophage), phage therapeutics

Antimicrobial resistance (AMR) remains one of the most critical threats to global health. In 2021, an estimated 4.71 million deaths were associated with bacterial AMR, including 1.14 million deaths directly attributable to AMR (Naghavi et al., 2024). Beyond its clinical impact, AMR also generates major burdens for healthcare systems and threatens food safety and environmental sustainability (Sharma et al., 2024). Despite scientific advances, efforts to curb AMR often fall short due to the complex ecological and evolutionary forces that drive resistance emergence and dissemination (Calero-Cáceres and Balcázar, 2024).

In this context, bacteriophage-based strategies have regained strong interest as alternatives and adjuncts to antibiotics, particularly as resistance increases to last-resort therapies (Pirnay, 2020; Calero-Cáceres and Balcázar, 2025). Although phage therapy is not yet routine in most settings, advances in molecular genetics and genomics now enable more rational selection, characterization, and engineering of phages, including approaches that minimize safety concerns by excluding undesirable genetic elements and improving standardization (Kakkar et al., 2024; Banerjee et al., 2025). Collectively, these developments are accelerating the translation of antibacterial interventions toward more personalized and targeted approaches. This Research Topic highlights advances in phage therapeutics across engineered approaches, clinical translation, microbiome-informed evaluation, and enabling infrastructure for therapeutic deployment.

Ludwig et al. explored engineered T7 lytic phages encoding antimicrobial peptides (AMPs) to enhance killing of multidrug-resistant *Escherichia coli*. The authors showed that incorporating AMP-encoding sequences can improve activity against both phage-susceptible and phage-resistant bacteria, and they further examined strategies to support peptide secretion and stability. While the work remains at the *in vitro* stage, it underscores the potential of AMP–phage platforms and highlights key optimization requirements, particularly around expression balance and genetic stability.

Alt et al. report a fracture-related infection in a severely injured patient with polymicrobial, multidrug-resistant pathogens, managed through surgical intervention, systemic antibiotics, and adjunct local phage therapy delivered via a hydrogel. Using molecular monitoring, the authors documented localized phage presence after administration without evident safety concerns, and the patient achieved infection resolution with successful reconstruction at follow-up. This case supports the feasibility of integrating phages into complex surgical infection management, particularly when conventional options are limited.

Wiese et al. describe that high-throughput screening *in-vitro* platform (“i-screen”) that evaluates phage activity against *Clostridium perfringens* within a complex chicken cecal microbiota, moving beyond simplified monoculture assays. In this microbiome-informed setting, a *C. perfringens*–specific phage suppressed pathogen growth with performance comparable to antibiotic benchmarks, while preserving microbiota structure more effectively and showing clear host specificity. The study provides a practical framework to prioritize candidate phages under ecologically relevant conditions, with implications for animal health and food production systems.

Finally, Topa-Pila et al. highlight phage biobanks as enabling infrastructure for precision phage deployment in the AMR era. They argue that scalable therapeutic implementation requires curated collections supported by standardized genomic and functional characterization, harmonized metadata, and interoperable workflows that accelerate phage–host matching. By positioning biobanks as decision-support systems within a One Health perspective, the paper emphasizes that governance, standardization, and data harmonization are essential requirements for coordinated clinical, veterinary, agricultural, and environmental applications.

While the promise of phage therapy is undeniable, its practical adoption necessitates addressing several challenges, including the optimization of phage cocktails for consistent and durable efficacy, clearer regulatory pathways, and scalable manufacturing with robust quality control (Morales and Hyman, 2025). In parallel, important knowledge gaps remain regarding phage–host dynamics in complex communities, the determinants of treatment success across different infection sites, and the evolutionary trajectories that shape both bacterial and phage resistance. Because genomics is increasingly central to phage discovery, characterization, and monitoring, it is also essential to recognize that methodological choices can introduce biases in phage sequencing and downstream interpretation, potentially limiting reproducibility and comparability across studies (Mora-Domínguez and Calero-Cáceres, 2025). Addressing these limitations will require harmonized protocols, transparent reporting standards, and standardized bioinformatic pipelines that support interoperable datasets and translation-ready evidence.

In conclusion, we hope this Research Topic reinforces phage therapeutics as a rapidly advancing and increasingly practical complement to antibiotics in the response to antimicrobial resistance. By bringing together studies spanning engineered phage–antimicrobial strategies, clinical implementation in complex infections, microbiome-informed evaluation platforms, and the development of phage biobanks as enabling infrastructure, this Research Topic highlights key directions shaping the field. Moving these advances toward broader uptake will require coordinated efforts to strengthen evidence generation and comparability, including standardized characterization frameworks, interoperable biobanking systems, and shared analytical workflows that support reliable phage–host matching across settings. Ultimately, global collaboration, interdisciplinary research, and innovation-driven translation will be essential to realize the full potential of phage-based interventions and to position them as a robust component of future AMR mitigation strategies.
